# Spatially Tunable Interfacial Ferroelectricity in Low-Symmetric WTe_2_

**DOI:** 10.1021/acs.nanolett.5c05610

**Published:** 2025-12-17

**Authors:** Yi-Cheng Chiang, Chun-An Chen, Che-Min Lin, Erh-Chen Lin, Hong-Sen Zhu, Po-Yen Liu, Yu-Ting Lin, Sheng-Hung Fan, Hung-Ju Tien, Chi Chen, Ying-Yu Lai, Hui Deng, Chia-Seng Chang, Hsin Lin, Tay-Rong Chang, Shang-Fan Lee, Yi-Hsien Lee

**Affiliations:** † Department of Materials Science and Engineering, 34881 National Tsing-Hua University, Hsinchu, 30013, Taiwan; ‡ 38017 Institute of Physics, Academia Sinica, Taipei, 115024, Taiwan; § Department of Physics, 34912 National Cheng Kung University, Tainan, 70101, Taiwan; ∥ National Center for Excellence in Quantum Information Science and Engineering, National Tsing-Hua University, Hsinchu, 30013, Taiwan; ⊥ Research Center for Applied Sciences, Academia Sinica, Taipei, 115024, Taiwan; # Center for Quantum Frontiers of Research and Technology (QFort), Tainan, 70101, Taiwan; 7 Physics Division, National Center for Theoretical Sciences, Taipei, 10617, Taiwan; 8 Department of Physics, 1259 University of Michigan, Ann Arbor, Michigan 48109-2122, United States

**Keywords:** interfacial ferroelectricity, WTe_2_, low symmetry, interlayer sliding, polarization switching

## Abstract

Interfacial ferroelectricity, recently discovered in van der Waals (vdW) materials, exhibits switchable dipoles at the interface. Most experiments are realized by stacking high-symmetry two-dimensional (2D) lattices in specific stacking configurations. A prototype based on a synthetic and low-symmetry 2D lattice is robust for switchable dipoles with broken symmetry at the interface. Here, we show that interfacial ferroelectricity can be spatially tunable by controlling the odd–even layer number in the synthetic low-symmetry lattice of 1T′-WTe_2_. A high ferroelectric transition temperature (*T*_C_) of >550 K is confirmed. The density functional theory (DFT) calculations indicate that interlayer sliding along the *b*-axis enables polarization switching of the interfacial dipoles. This study moves a significant step toward spatially tunable interfacial ferroelectricity.

## Graphical abstract

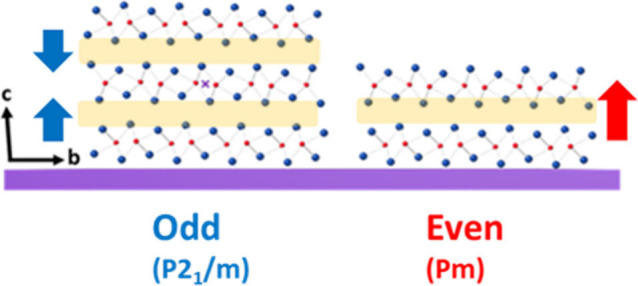

Interfacial ferroelectricity, a new type of switchable dipole, has recently been realized in stacked bilayer^1–15^ or multilayer^1,3,4,7,8,10,11,16–20^ vdW materials with specific stacking. In contrast to conventional ferroelectrics, uncompensated charge transfer or interlayer interactions in the stacked 2D lattices enable a novel charge distribution at the interface with unique switchable dipoles. In reported papers, the samples were mainly prepared based on vdW crystals of high-symmetry lattices using the tear-and-stack method. However, specific stacking configurations and ideal interface quality are critically required to enable the interfacial ferroelectricity. This raises considerable efforts for diverse artificially stacked 2D lattices and spatially resolved measurements to verify stacking configurations.

Ferroelectricity in the low-symmetry 2D lattices was discovered with the exfoliated WTe_2_ of the *T*_d_ phase.^1,2,16^ Recently, theoretical prediction suggests a possible interlayer sliding in *T*_d_-WTe_2_.^21,22^ For better understanding and manipulation of the interfacial ferroelectricity, a stable 1T′ phase of the scalable WTe_2_ is selected for spatially tuned inversion symmetry. The interfaces among the odd–even layer regions offer a significant degree of freedom to study the layer-dependent dipoles at the interfaces. Compared to the 1T′-WTe_2_ synthesized by chemical vapor deposition (CVD), a stable *T*_d_ phase commonly appears in the exfoliated WTe_2_, and its ferroelectricity always appears from the bilayer to higher thickness. A tunable symmetry and scalable prototype are essential for the possible application and better manipulation in interfacial ferroelectricity.

Here, we demonstrate the scalable and spatially controllable interfacial ferroelectricity using the representative low-symmetry 2D lattices of the 1T′-WTe_2_. Optimized growth reactions in CVD enable precise control of the phase and thickness of synthetic WTe_2_ in a large area.

Controlling the odd–even layer numbers, the synthetic 1T′-WTe_2_ exhibits a uniform layer-dependent symmetry and ferroelectric (FE) response. A high *T*_C_ above 550 K was evaluated by using cycling and variable-temperature second harmonic generation (SHG) measurements. The DFT calculations indicate that interlayer sliding along the *b*-axis enables polarization switching of the interfacial dipoles in the synthetic low-symmetry 2D crystals. It is experimentally and theoretically demonstrated that the 1T′-WTe_2_ is a robust prototype for scalable and spatially tunable interfacial ferroelectricity.

## Results and Discussion

### Odd–Even Layer-Dependent Symmetry in the Synthetic 1T′-WTe_2_

A broken symmetry of the 2D lattice can be effectively achieved by controlling the crystalline phase and stacking of the vdW materials. Figure 1a illustrates the odd–even layer-dependent symmetry of the 1T′-WTe_2_ (see Figure S1 for more information on crystal structure and Table S1 for schematic comparison of the difference between *T*_d_- and 1T′-WTe_2_). The lattice with odd-layer numbers belongs to space group *P*2_1_/*m* (C_2h_^2^ in Schönflies notation), while that with even-layer numbers belongs to space group *Pm* (C_s_^1^). Space group *P*2_1_/*m* exhibits a 2-fold rotation by the screw axis 2_1_ and a mirror plane perpendicular to the *c*-axis, leading to the lattice with odd-layer numbers always showing an inversion symmetry center at the middle layer (violet cross mark as indicated). In contrast, a broken inversion symmetry appears in even-layer numbers with vdW monoclinic stacking because screw rotation symmetry along the *c*-axis is broken in the symmorphic space group *Pm*. The WTe_2_ crystal is a representative low-symmetry vdW material with a stable *T*_d_ phase and anisotropic properties. To achieve the interfaces at layer-dependent symmetry, the 1T′ phase of the WTe_2_ with tunable thickness was synthesized by CVD with the KCl promoters (Methods). More details on the chemical configuration of the synthetic 1T′-WTe_2_ are shown in Figure S2. A broken inversion symmetry is induced at the 1T′-WTe_2_ of the even-layer numbers, enabling a uniform layer-dependent symmetry contrast in a large area. As shown in Figure 1b, the as-grown 1T′-WTe_2_ exhibits clear odd–even layer-dependent contrast, and its layer number is precisely identified by atomic force microscopy (AFM). The two representative crystalline symmetries for the odd- and even-layer 1T′-WTe_2_ (Figure S3a) are confirmed with selected area electron diffraction (SAED) (Figure 1c,d) and fast Fourier transform (FFT) patterns (Figure S3b,c) using high-angle annular dark-field scanning transmission electron microscopy (HAADF-STEM) and high-resolution transmission electron microscopy (HRTEM). In the SAED of the two representative symmetries, the {120} planes appear in the odd-layer region and disappear in the even-layer region, consistent with the simulated diffraction patterns (Figure 1c,d). The missing diffraction spots in Figure 1d indicate a broken inversion symmetry of the 1T′-WTe_2_ with even-layer numbers. Different from the 2H-MoS_2_ and the transition metal dichalcogenides (TMDs) with space group *P*6_3_/*mmc* (D_6h_), the WTe_2_ exhibits anisotropic physical properties and low-symmetry lattices with a one-dimensional (1D) distortion.^23–26^

**Figure 1:**
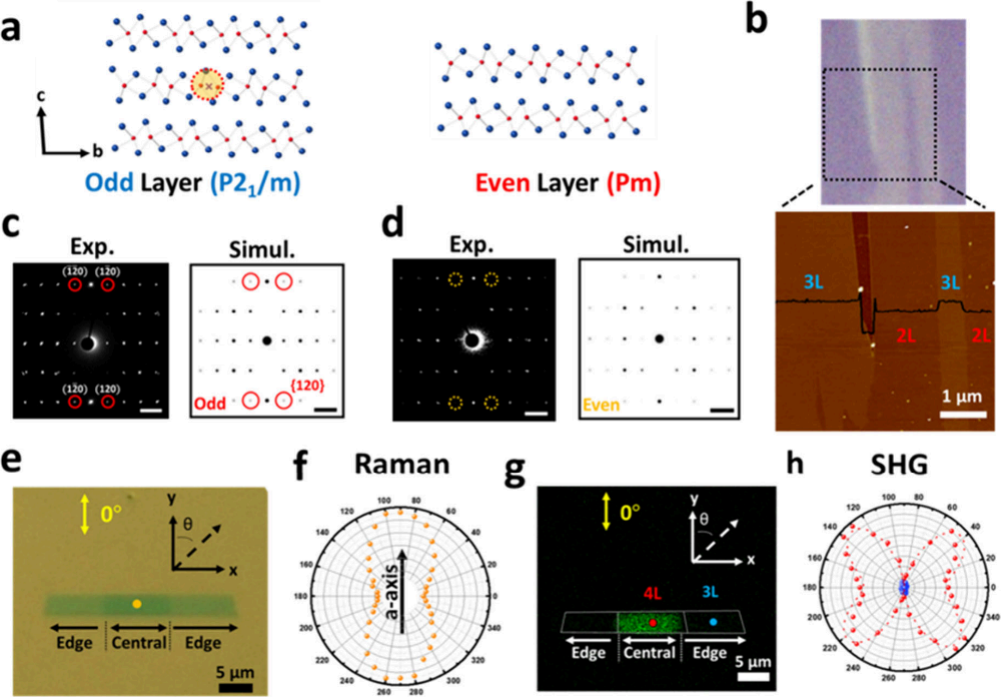
Odd–even layer-dependent symmetry in the synthetic 1T′-WTe_2_. (a) Schematic illustration of the 1T′-WTe_2_ crystal structures with odd- and even-layer numbers. The violet cross mark indicates an inversion symmetry center. (b) Optical image and surface topography of the thickness-tuned 1T′-WTe_2_. The AFM height profile shows the distribution of the bilayer (∼1.5 nm) and trilayer (∼2.1 nm) regions. (c, d) SAED pattern and its simulated diffraction pattern of the (c) odd- and (d) even-layer 1T′-WTe_2_. The diffraction spots from {120} planes were highlighted as red circles in (c). Yellow circles in (d) indicate the absence of diffraction spots from {120} planes. The scale bars represent 2 1/nm. (e) Optical image of the single crystalline few-layer 1T′-WTe_2_. (f) Polar plot with the copolarized scheme for the A_1_ Raman mode at the central region (the yellow spot). The arrow indicates the crystalline orientation of the *a*-axis. (g) SHG image of the same flake in (e). (h) Polar plot with the copolarized scheme for the SHG at the central region (the red spot) and edge region (the blue spot).

Nondestructive verification approaches, including polarization-resolved Raman and SHG spectroscopies, were adopted to study the layer-dependent symmetry, combined with the anisotropic crystal orientation effects in the WTe_2_. A confocal microscope system performed the polarization-resolved Raman spectroscopy with a 532 nm pumping laser. Co-polarized configurations were constructed to reveal the polarization response of the Raman signals by inserting an analyzer before the spectrometer. According to the previous work,^25^ the A_1_ Raman mode at 163 cm^–1^ achieves its maximum as the pumping laser is polarized along the *a*-axis, identifying the crystal orientations. Figure 1e shows an optical image of a single-crystalline 1T′-WTe_2_ with a thickness contrast for the odd–even layer dependence. The central and the edge regions in the belt-shaped single-crystalline domain are tetra- and trilayers, respectively. In the angle-resolved intensity evolution shown in Figure 1f, the A_1_ mode of the central region (the yellow spot in Figure 1e) respectively reaches a maximum and minimum as the laser is polarized at the experimental coordinate of the *x*-axis and *y*-axis shown in Figure 1e, confirming the anisotropic properties of the 1T′-WTe_2_ with the crystalline *a*-axis parallel to the *x*-axis.

As for polarization-resolved SHG spectroscopy, an 850 nm Ti-sapphire laser with a pulse width of 75 fs is utilized as the fundamental wave in nonlinear wavelength conversion. Figure 1g shows the SHG intensity (λ = 425 nm) mapping of the same flake in Figure 1e with laser polarization along the *y*-axis of the experimental coordinate. It is observed that SHG emission is much stronger at the tetralayer central region of the flake than at the trilayer edge region. The region of the even-layer numbers exhibits an intense SHG emission, while the SHG signal disappears in that of the odd-layer numbers. The angular evolutions of SHG emission at the central region (the red spot in Figure 1g) and edge region (the blue spot in Figure 1g) are plotted in Figure 1h. In the edge region, the SHG signal confirms a preserved inversion symmetry of the 1T′-WTe_2_ in odd-layer numbers. In the central region as a clear contrast for odd–even layer-dependent symmetry effects, a strong angular-dependent SHG signal appears with the butterfly-like intensity evolution being well-fitted by the nonlinear response of the C_S_ point group notation,^27^ proving the broken inversion symmetry of the 1T′-WTe_2_ in even-layer numbers. The highly reduced SHG intensities along the *a*-axis can also clearly verify the crystallographic orientation of the single-crystalline 1T′-WTe_2_. Results shown in Figure 1g,h agree well with those of transmission electron microscopy (TEM) studies. They are distinct from the previous work,^1,2,16^ where structural symmetry is independent of the odd–even layer numbers.

### Spatially Tunable Domains of the Interfacial Ferroelectrics

To further study the ferroelectricity in the 1T′-WTe_2_ of the odd–even layer-dependent symmetry, piezoresponse force microscopy (PFM) is adopted to probe the local FE properties with precise identification of the layer numbers in each single-crystalline domain of the scalable CVD samples. The transferred CVD samples with an ultraclean surface and interface quality are prepared on conducting substrates by the water-assisted process (Methods). Along the green line, the AFM height profile (Figure 2a) highlights three representative regions in the odd–even layer 1T′-WTe_2_ (red: 4L, blue: 5L, and violet: substrate). PFM enables simultaneous verification of the spatial distribution of the layer and the switchable dipoles at the nanoscale. Controlling the spatially distributed domains with specific odd–even layer numbers induces a uniform FE response. It is observed in the regions of the even-layer 1T′-WTe_2_ (tetralayer in red), while disappears in that of the odd-layer regions (pentalayer in blue), which illustrates spatially tunable properties and is consistent with the symmetry-dependent optical properties as shown in Figure 1. In the literature, the origin of the dipoles in most vdW FE is not attributed to interfacial interactions, but the polarization is determined based on the superposition of the dipoles in the entire crystal structure; for example, an opposite polarity of the intralayer dipoles would cancel the polarization with even-layer numbers,^28,29^ which is a clear contrast to the polarization from the interfacial dipoles for interfacial ferroelectrics. Figure 2b shows a representative PFM phase image after probing from the tip under direct current (DC) bias, confirming the FE signature of the WTe_2_ because switchable domains and the response under DC bias are signatures for stable ferroelectricity. Three square patterns with ±12 V_DC_ from the tip in the bilayer 1T′-WTe_2_ (Figure 2b) are 5 μm by 5 μm, 3.5 μm by 3.5 μm, and 2 μm by 2 μm, respectively. The local phase hysteresis and amplitude loops, indicating a 180° phase reversal and a characteristic butterfly-shaped amplitude response, are shown in Figure S4. A uniform and clear antiparallel contrast in even-layer regions of the 1T′-WTe_2_ suggests robust switchable dipoles of the even-layer 1T′-WTe_2_.

**Figure 2:**
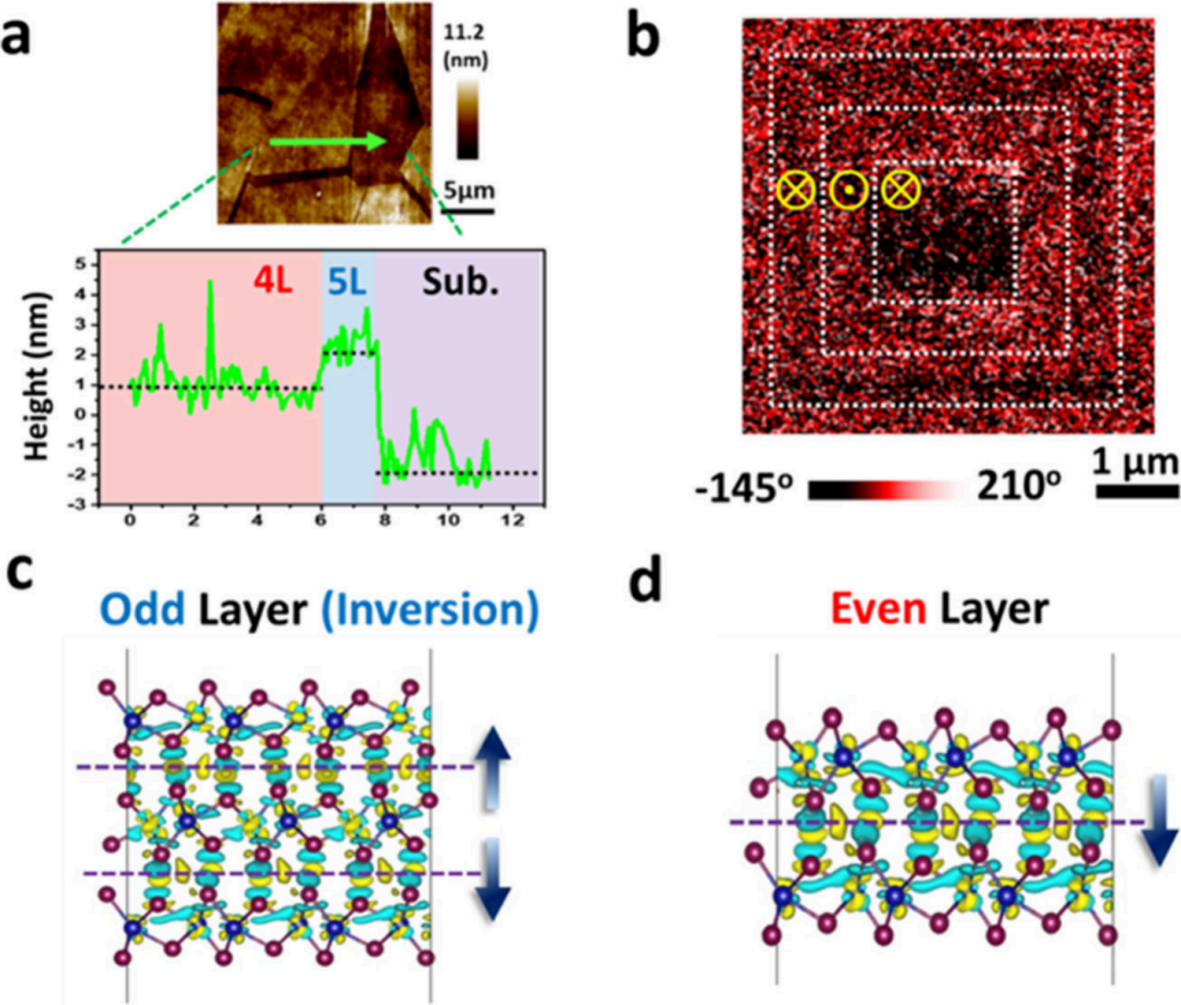
Spatially tunable domains of the interfacial ferroelectrics. (a) Surface topography with the height profile of the layer-dependent 1T′-WTe_2_. The green arrow indicates the scanning direction. (b) Patterned domains in the bilayer 1T′-WTe_2_ illustrate the switchable interfacial dipoles. (c, d) Charge density plot and the interfacial dipoles of the tri- and bilayer 1T′-WTe_2_, respectively. The blue arrows indicate the direction of the polarization at the interface.

Figure 2c,d illustrate the calculated real-space charge density distributions of odd- (trilayer) and even-layer (bilayer) WTe_2_, respectively. The isosurface coloration in yellow and green signifies the variations in charge density compared to individual monolayers of WTe_2_, indicating augmentation and reduction in multilayer WTe_2_. Notably, we observe the concentration of local dipoles primarily at the interface between the two layers of WTe_2_, with their charge clouds directed toward the Te atoms along the *z*-axis, providing essential theoretical evidence of interfacial dipoles. In the trilayer slab model, the charge density distribution between the first two layers resembles that of the bilayer model. In contrast, the second and third layers exhibit opposing density distributions due to the spatial inversion symmetry of the slab structure. Consequently, trilayer WTe_2_ demonstrates zero net polarization across the entire system. The polarization direction of each local dipole in odd-layer WTe_2_ is 180° opposite that of its adjacent dipole, resulting in mutual cancellation. At the same time, an additional charge density distribution is evident in the interstitial spaces between interface Te atoms. It contributes to the net polarization in even-layer WTe_2_, which is well-matched to the experimental observations of odd–even layer-dependent ferroelectricity in 1T′-WTe_2_ (Figure 2b). Further calculations reveal that the intensity of polarization remains consistent regardless of thickness in even-layer numbered models.

### Temperature-Dependent Symmetry in the Even-Layer 1T′-WTe_2_

To gain more insights into the interfacial ferroelectricity in the 1T′-WTe_2_, cycling and variable-temperature SHG measurements are performed to study the temperature dependencies and *T*_C_ of the even-layer 1T′-WTe_2_. The measurements are carried out in a vacuum (10^–2^ Torr) chamber, and a single-crystalline 1T′-WTe_2_ flake (Figure 3a) on a Si_3_N_4_ substrate is adopted to avoid possible oxidation. The SHG intensity mapping (λ = 425 nm) shown in Figure 3b indicates the even- and odd-layer regions. A strong SHG emission from the even-layer regions (tetra- and hexalayer) suggests a broken inversion symmetry. At the same time, the SHG signals disappear in the odd-layer regions (tri- and heptalayer) due to the preserved inversion symmetry. Additional SHG measurements of layer-dependent inversion symmetry are presented in Figure S6. In the even-layer regions of WTe_2_, a temperature-dependent SHG intensity is observed in every heating and cooling cycle (Figure 3c,d). On the contrary, odd-layer regions of the WTe_2_ show no dependency (Figure S5). With the cycling measurements, the temperature-dependent SHG could be highly reversible, and an intensity reduction of ∼60% is observed when the temperature increased from 60 to 280 °C, indicating a high temperature for the ferroelectric–paraelectric (FE-PE) phase transition. In the literature, the record for the highest *T*_C_ of the *T*_d_-WTe_2_ is ∼77 °C (350 K),^1^ while the even-layer 1T′-WTe_2_ in this study exhibits a *T*_C_ above 280 °C, suggesting a robust sample quality. Notably, due to the temperature-control limitations of our variable-temperature measurement setup, the *T*_C_ could only be determined up to approximately 280 °C. The unsaturated curves in Figure 3c,d suggest that the actual *T*_C_ may be higher. Calculations for the charge density plot and the interfacial dipoles of tetra- and hexalayer 1T′-WTe_2_ are shown in Figure 3e,f, respectively. In the tetralayer (hexalayer) configurations, the polarizations at the interface cancel each other for net polarization. The noncentrosymmetry in even-layer 1T′-WTe_2_ originates from the symmetry breaking of the screw rotation along the *c*-axis, which induces local dipoles at the interfacial regions between adjacent layers rather than within the bulk (Figure 2d). In multilayer configurations, these interfacial dipoles interact such that neighboring dipoles largely cancel each other, leaving one uncompensated interfacial polarization that gives rise to the observed net FE polarization (Figure 3e,f). Therefore, the crystallographic symmetry breaking provides the structural basis for dipole formation, while the incomplete cancellation of interfacial polarizations explains the emergence of net ferroelectricity in even-layer 1T′-WTe_2_.

**Figure 3:**
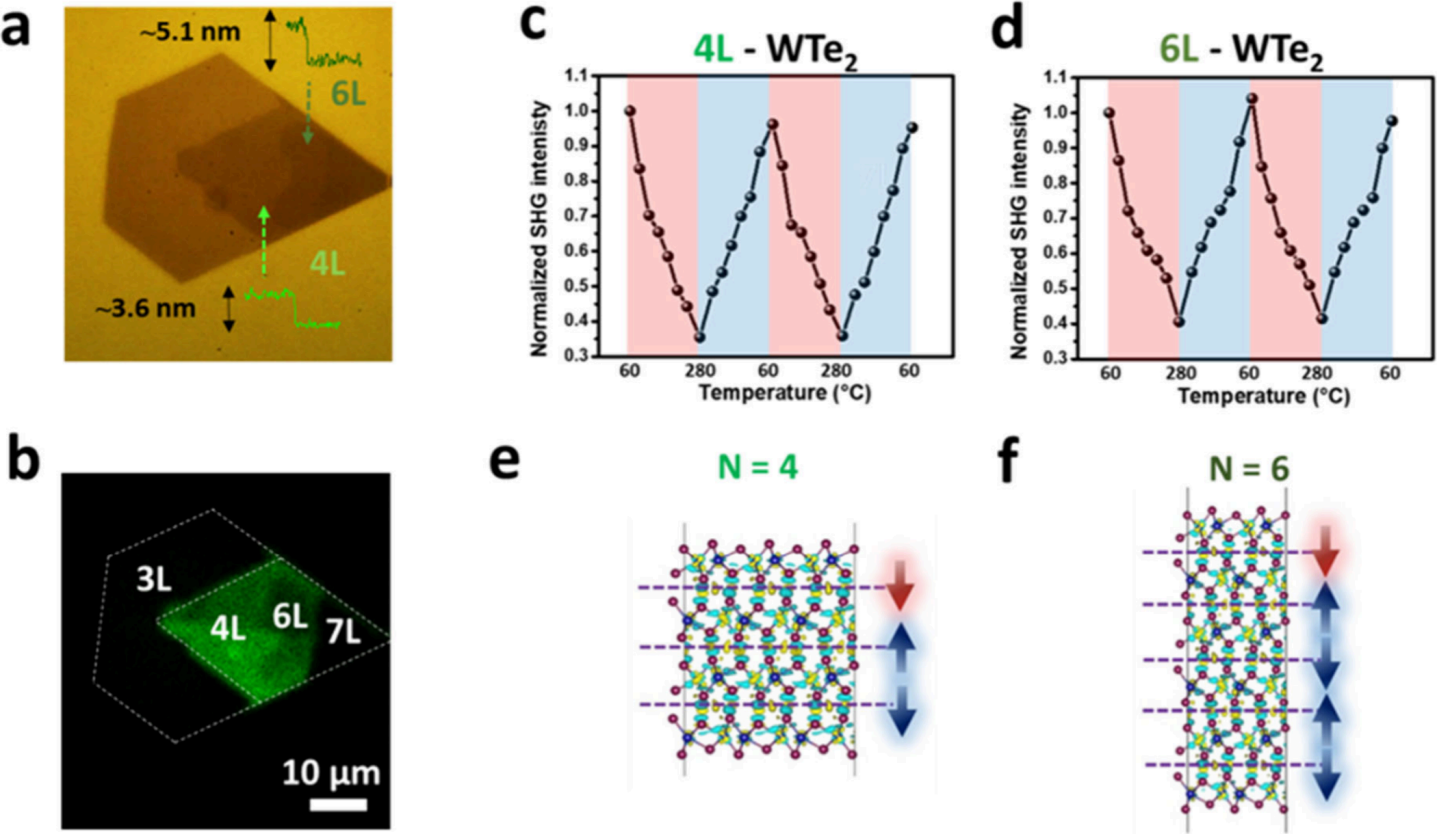
Temperature-dependent symmetry in the even-layer 1T′-WTe_2_. (a, b) Optical and SHG images of the few-layer 1T′-WTe_2_, respectively. Insets in (a): the AFM height profiles confirm the two different even-layer numbers with the tetra- (green: ∼3.6 nm) and hexalayer (dark green: ∼5.1 nm) regions. (c, d) Normalized SHG intensity with two variable-temperature cycles in the tetra- (c) and hexa- (d) layer 1T′-WTe_2_. (e, f) Charge density plot and the interfacial dipoles of the tetra- (e) and hexa- (f) layer 1T′-WTe_2_. The blue arrows cancel each other, and the remaining red ones indicate the direction of net polarization.

### Interlayer Sliding of the Layer-Dependent Interfacial Ferroelectricity

Our DFT calculations reveal that polarization arises from the charge disparity of dipoles at the interface of WTe_2_ layers. This discovery suggests that adjusting the alignment of two WTe_2_ layers can manipulate local dipoles, thus controlling the FE polarization (*P*_2D_). Consequently, we shifted the relative positions of the two layers in bilayer 1T′-WTe_2_, as shown in Figure 4 and Figure S7. Our findings (inset of Figure 4a) indicate that the intensity of polarization varies linearly. The polarization direction flips when the top layer slab is shifted along the *y*-axis, i.e., the *b*-axis in Figure 1a. Through structure optimization, we identified two energy minimum structures occurring at positions Δ*y* = ±2.8%, where Δ*y* represents the displacement ratio in the *y*-axis relative to the structure with *P*_2D_ = 0 (Figure 4a). The polarization strength at these two energy minimum structures is found to be *P*_2D_ = 0.12 pC/m and *P*_2D_ = – 0.12 pC/m, respectively, demonstrating that the direction of polarization can be switched by sliding between the two layers of 1T′-WTe_2_. Notably, the energy barrier required for polarization switching of interfacial dipoles is minimal (∼0.1 meV), indicating that interlayer sliding is energetically favorable. In our work, *P*_2D_ and energy barrier are comparable to the values for bilayer *T*_d_-WTe_2_.^21,22^ To further explore the origin of polarization flipping, charge density plots corresponding to Δ*y* = 2.8%, 0%, and −2.8% are separately depicted in Figure 4b–d, respectively. At Δ*y* = 2.8%, polarization is induced by additional charge density in the interstitial spaces between interface Te atoms (Figure 4b). In contrast, at Δ*y* = 0%, the Te atoms of the top and bottom layers are perfectly aligned, resulting in the cancellation of each local dipole with its neighboring dipole, yielding zero polarization (Figure 4c). When the energy barrier is overcome by interlayer sliding, the charge density in the interstitial space between Te atoms redistributes, bringing the charges closer to the bottom layer compared to Δ*y* = 2.8%, thereby reversing the direction of polarization (Figure 4d). Calculation for one-dimensional (1D) layer-averaged differential charge density elucidates the microscopic origin of the interfacial ferroelectricity (Section S8), while the even–odd layer-number-dependent polarization illustrates the symmetry-driven origin of interfacial dipoles (Section S9).

**Figure 4:**
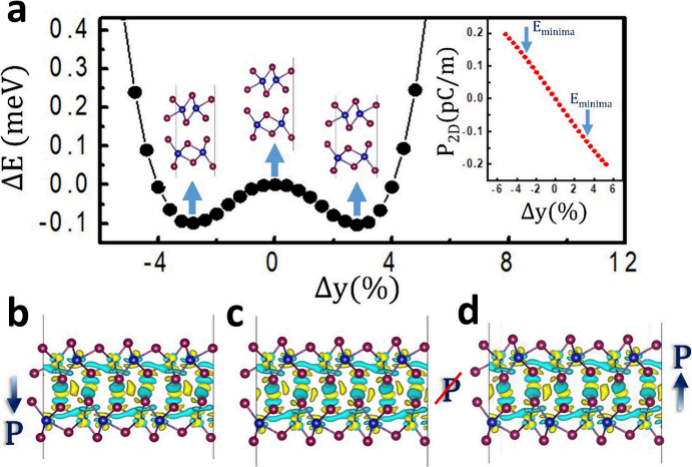
Interlayer sliding of the layer-dependent interfacial ferroelectricity. (a) Energy profile as a function of top layer shift Δ*y*. Inset in (a): FE polarization as a function of top layer shift Δ*y*. (b–d) Charge density plot of the two FE structures (b, d) and the nonpolar structure (c) of bilayer 1T′-WTe_2_.

## Conclusions

Spatially tunable interfacial ferroelectricity is achieved with the *T*_C_ above 550 K in the CVD-grown 1T′-WTe_2_. The odd–even layer-dependent symmetry of the low-symmetry 1T′-WTe_2_ enables the spatial distribution of the interfacial ferroelectricity. The layer-dependent symmetry, realized only in the low-symmetric 2D lattices, will induce new physical phenomena and properties. The DFT calculations confirm that switchable interfacial dipoles in even-layer 1T′-WTe_2_ are attributed to interlayer sliding at the *b*-axis. The synthetic 1T′-WTe_2_ suggests a prototype for controllable interfacial ferroelectricity.

## Supplementary Material



## Notes

Crystal structure of synthetic 1T′-WTe_2_; chemical configurations of WTe_2_; spatially dependent crystal structure of odd- and even-layer 1T′-WTe_2_; local PFM measurements of even-layer 1T′-WTe_2_; cycling of variable-temperature SHG for odd–even-layer dependence; additional SHG data for layer-dependent inversion symmetry; DFT calculations for interlayer sliding; 1D layer-averaged differential charge density; DFT calculations for layer-number-dependent polarization; detailed experimental methods (PDF)
