# Supplementary material for: Spatially Tunable Interfacial Ferroelectricity in Low-Symmetric WTe2
Source: Nano Lett. 2025 Dec 17;26(2):864–70. doi: 10.1021/acs.nanolett.5c05610 (PMC12833872; doi:10.1021/acs.nanolett.5c05610)
Supplement: Supplementary file 1 [file nl5c05610_si_002.docx]

Supplementary Information for

**Spatially-tunable interfacial ferroelectricity in the Low Symmetric WTe_2_**

Yi-Cheng Chiang^1^†, Chun-An Chen^1,2^†, Che-Min Lin^3^†, Erh-Chen Lin^1^†, Hong-Sen Zhu^1,4^, Po-Yen Liu^,1,4,8^, Yu-Ting Lin^1^, Sheng-Hung Fan^1^, Hung-Ju Tien^3^, Chi Chen^5^, Ying-Yu Lai^1,8^, Hui Deng^8^, Chia-Seng Chang^2^, Hsin Lin^,2^, Tay-Rong Chang^3,6,7^, Shang-Fan Lee^2^, Yi-Hsien Lee^1,4^*

†These authors contribute equally to this work

*Corresponding Author: E-mail: [yhlee.mse@mx.nthu.edu.tw](mailto:yhlee.mse@mx.nthu.edu.tw) (Y.-H.L.)

**Contents**

**S1 Crystal structure of the synthetic 1T’-WTe_2_**

**S2 Chemical configurations of the WTe_2_**

**S3 Spatially dependent crystal structure of the odd- and even-layer 1T’-WTe_2_**

**S4 Local PFM measurements of the even-layer 1T’-WTe_2_**

**S5 Cycling of variable temperature SHG for the odd-even-layer dependence**

**S6** **Additional SHG Measurements of layer-dependent inversion symmetry**

**S7 Theoretical DFT calculations for interlayer sliding**

**S8 Plot of 1D layer-averaged differential charge density**

**S9 DFT calculations for layer-number-dependent polarization**

**S10 Methods**

**Table S1 Schematic comparison of difference between T_d_- and 1T’-WTe_2_**

**Table S2 Crystallographic data for the 1T’-WTe_2_**

**References**

**S1. Crystal structure of the synthetic 1T’-WTe_2_.**

HAADF-STEM is performed to confirm the crystal structure of the synthetic WTe_2_. In **Figure S1a**, the buckled zigzag W-W chains and distorted layers of the Te atoms are observed, consistent with the structural model of the 1T’-WTe_2_ (**Figure S1b**). The lattice parameters along the a-axis and b-axis extracted from **Figure S1a** are about 3.450 Å and 6.270 Å, respectively, which agree with those of the bulk WTe_2_ crystal (**Table S2**).

**
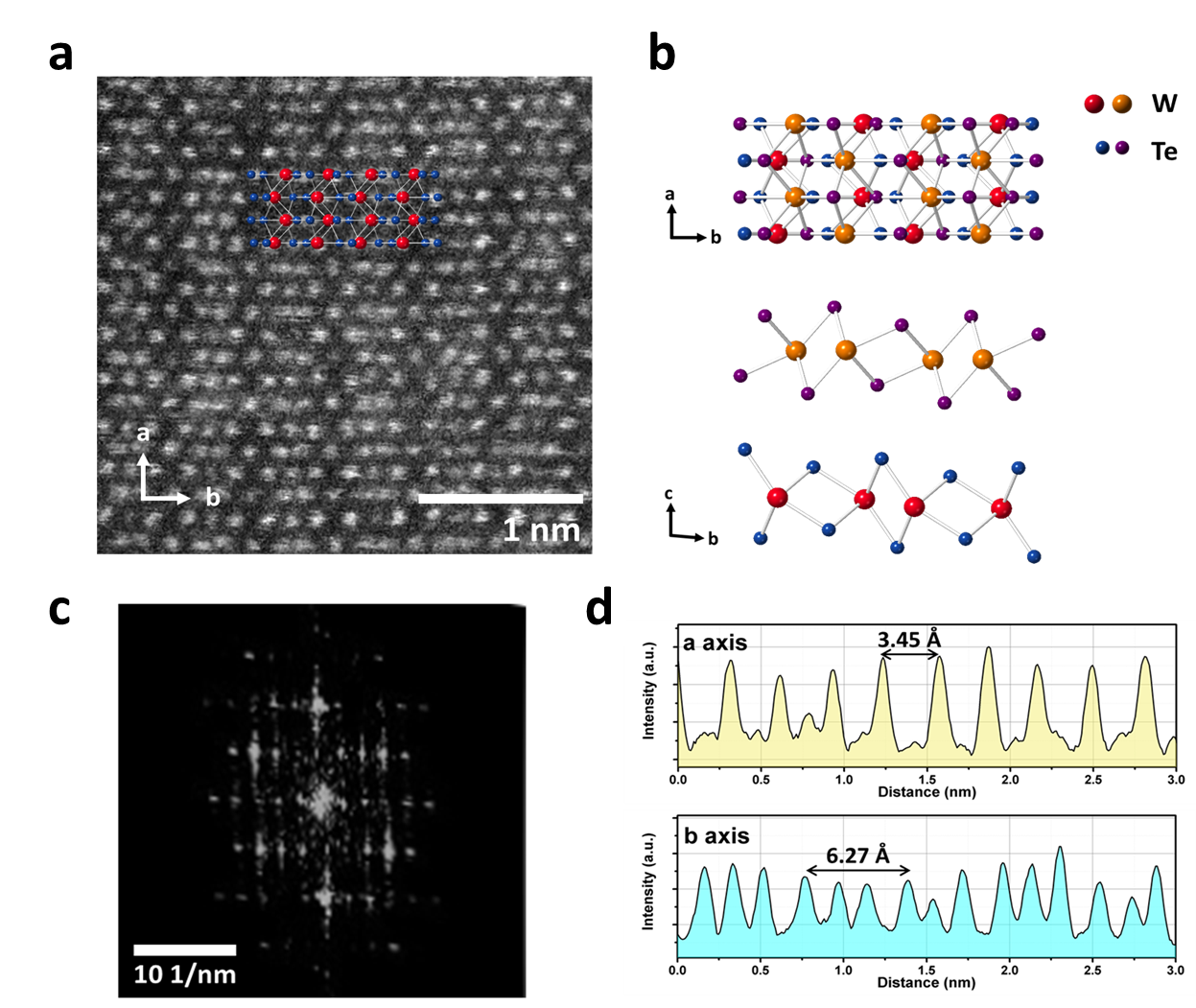
**

**Figure S1.** Crystal structure of the synthetic 1T’-WTe_2_. (a) HAADF-STEM image. The red spheres represent the W in the zigzag chains along a-axis, while the blue spheres represent the Te. (b) Top and side views of the bilayer structural models for octahedral coordinated monoclinic 1T’-WTe_2_. (c) The corresponding FFT image shows a rectangular spot pattern. (d) The line profiles along a- and b-axis confirm the lattice parameters (a = 3.450 Å and b = 6.270 Å).

**S2. Chemical configurations of the WTe_2_.**

To determine the chemical configurations and stoichiometry of the synthetic WTe_2_, x-ray photoelectron spectroscopy (XPS) are performed in ULVAC-PHI PHI 5000 Versaprobe II with an ultrahigh vacuum (UHV) chamber at a base pressure of ~5x10^-8^ Pa and an x-ray source of monochromatic Al Kα (1486.6 eV). To eliminate possible contaminations, the synthetic WTe_2_ films were transferred onto fresh SiO_2_/Si substrates with an ultra-clean water-assisted method. Core level peaks of the Te (3d_3/2_ and 3d_5/2_) are located at 583.1 eV and 572.7 eV, respectively (**Figure S2a**). The W peaks (4f_5/2_ and 4f_7/2_) are at 33.7 eV and 31.5 eV, respectively (**Figure S2b**). The Te/W ratio is estimated to be 2.08, confirming that the synthetic 1T’-WTe_2_ films are stoichiometric.

**
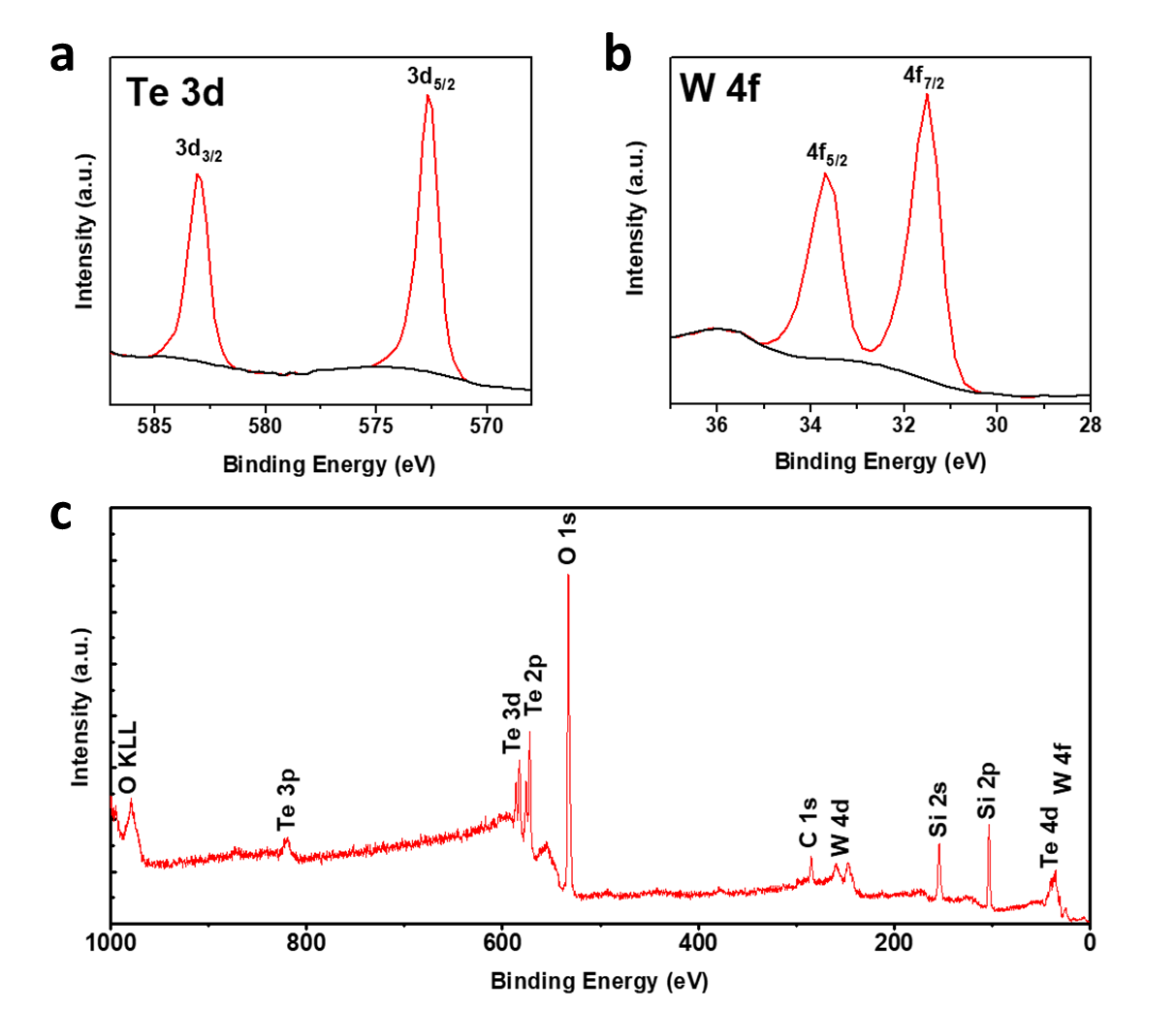
**

**Figure S2.** Chemical configurations of the WTe_2_. (a,b) XPS fine scan spectra of Te 3d (a) and W 4f (b). (c) XPS survey spectrum which demonstrates the high grade of our WTe_2_ films.

**S3. Spatially dependent crystal structure of the odd- and even-layer 1T’-WTe_2_.**

**Figure S3** indicates the spatial distribution and the crystal structure of the odd- (red square) and even- (yellow square) layer regions in the 1T’-WTe_2_. The symmetry of the samples with different layer numbers is identified using the SAED patterns (**Figure 1c,d**) and the FFT images (**Figure S3b,c**) from HRTEM.

**
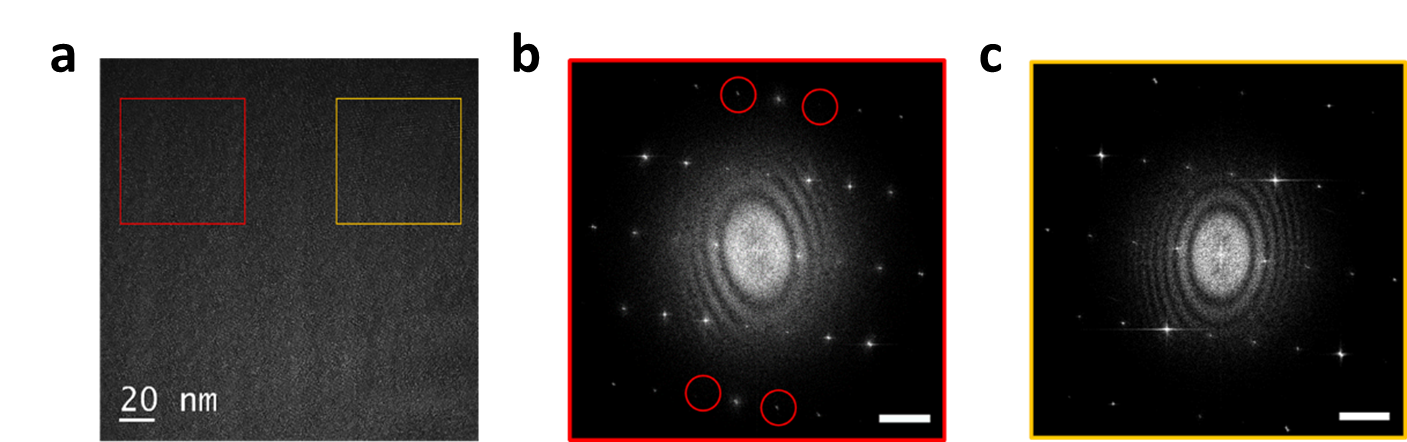
**

**Figure S3.** Spatially dependent crystal structure of the odd- and even-layer 1T’-WTe_2_. (a) HRTEM image of WTe_2_ with the junction between odd- (red square) and even- (yellow square) layer numbers. (b,c) The FFT images of the odd- (b) and even- (c) layer regions marked in (a). The diffraction spots from {120} planes featuring odd-layer numbers are highlighted as red circles. The white scale bars represent 2 1/nm in (b,c).

**S4. Local PFM measurements of the even-layer 1T’-WTe_2_.**

**
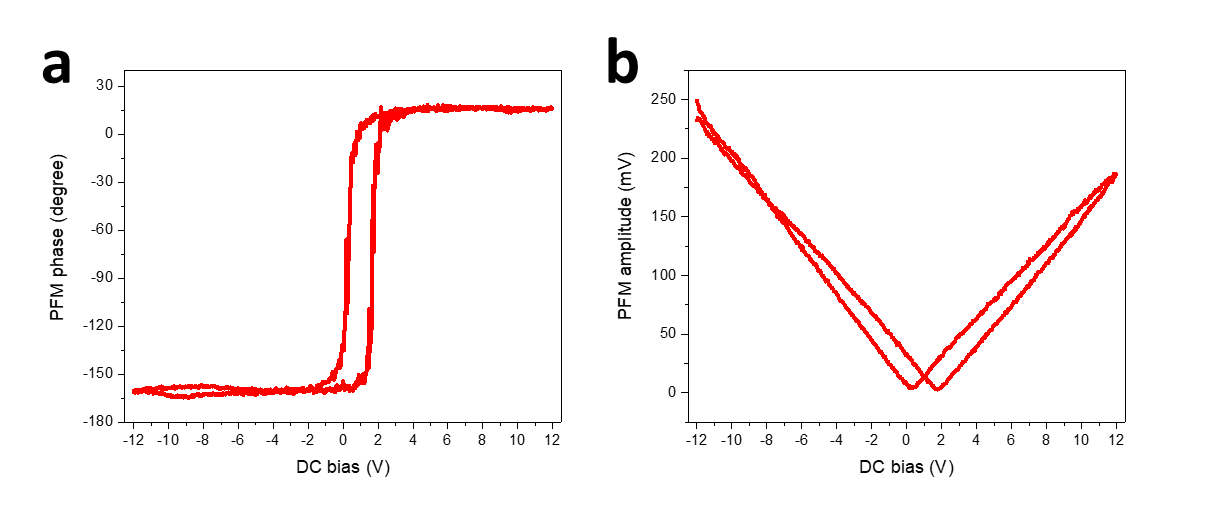
**

**Figure S4.** Local PFM measurements of the even-layer 1T’-WTe_2_. (a) The local phase hysteresis loop. (b) The local amplitude loop.

**S5. Cycling of variable temperature SHG for the odd-even-layer dependence.**


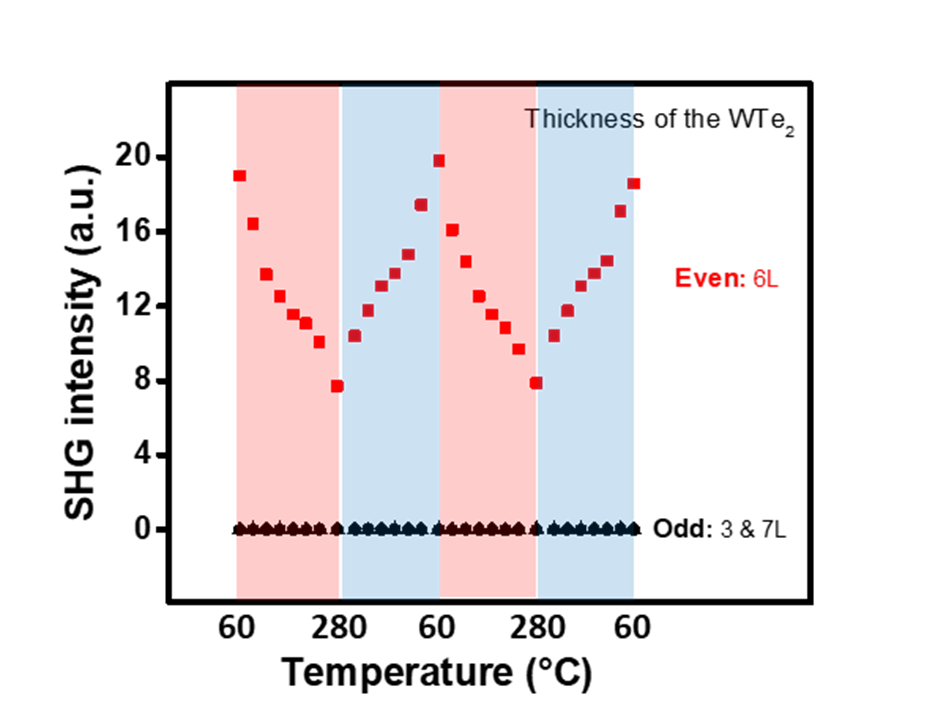


**Figure S5.** Cycling of variable temperature SHG for the odd-even-layer dependence. SHG intensity with two variable temperature cycles in the tri-, hexa-, and hepta-layer 1T’-WTe_2_ (**Figure 3b**).

**S6 Additional SHG Measurements of layer-dependent inversion symmetry**

To better demonstrate the layer-dependent properties and complement the data presented in main text (Figure 3: 3L, 4L, 6L, and 7L), we have further included SHG measurements on 2L and 5L regions. The observed contrast again follows the characteristic odd/even-layer dependence of the SHG intensity, providing additional confirmation of the broken inversion symmetry in even-layer 1T′-WTe₂. This reproducible trend across multiple samples strongly supports the layer-dependent ferroelectric behavior in 1T′-WTe₂.

**
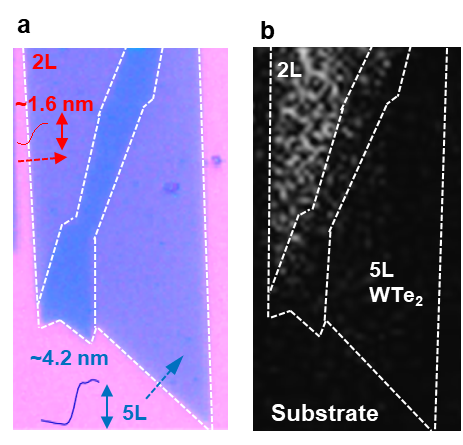
**

**Figure S7.** AFM height profile, (a) Optical microscopy image and (b) SHG intensity mapping of the odd (5L) and the even (2L) layer 1T’-WTe_2_.

**S7. Theoretical DFT calculations for interlayer sliding.**

The use of different calculation methods may cause variations in the energy barrier. To verify the impact of these methods, we employed different dispersion corrections and compared the energy versus in-plane sliding relationship, as shown in **Figure S6**.


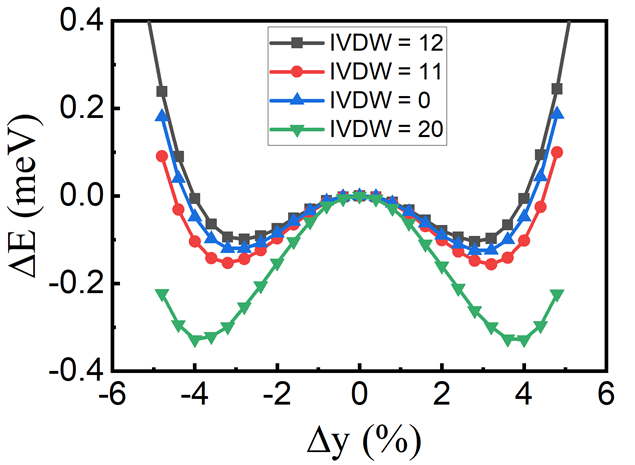


**Figure S7.** Theoretical DFT calculations for interlayer sliding. The variation in energy with in-plane sliding $\Delta$y (%) under different dispersion correction conditions.

**S8 Plot of 1D layer-averaged differential charge density**

To further elucidate the microscopic origin of the interfacial ferroelectricity, we computed the one-dimensional layer-averaged differential charge density, Δρ(z), for the two opposite sliding configurations. As shown in the newly added figure, both polarization states exhibit a pair of antisymmetric charge accumulation and depletion peaks localized at the interlayer region. The sign of Δρ(z) reverses upon switching the sliding direction, demonstrating that the interlayer charge transfer is fully inverted when the system transitions from one polarization state to the other. The amplitude of these peaks reaches approximately ±0.05–0.07 e/Å, indicating a robust interfacial dipole moment consistent with the DFT-calculated 2D polarization of ±0.12 pC/m. These results provide unambiguous layer-resolved evidence that the ferroelectric polarization in 1T′‑WTe₂ originates from sliding-induced charge disproportion at the interface, and that the direction of polarization is determined by the direction of charge transfer between the two adjacent layers.

**
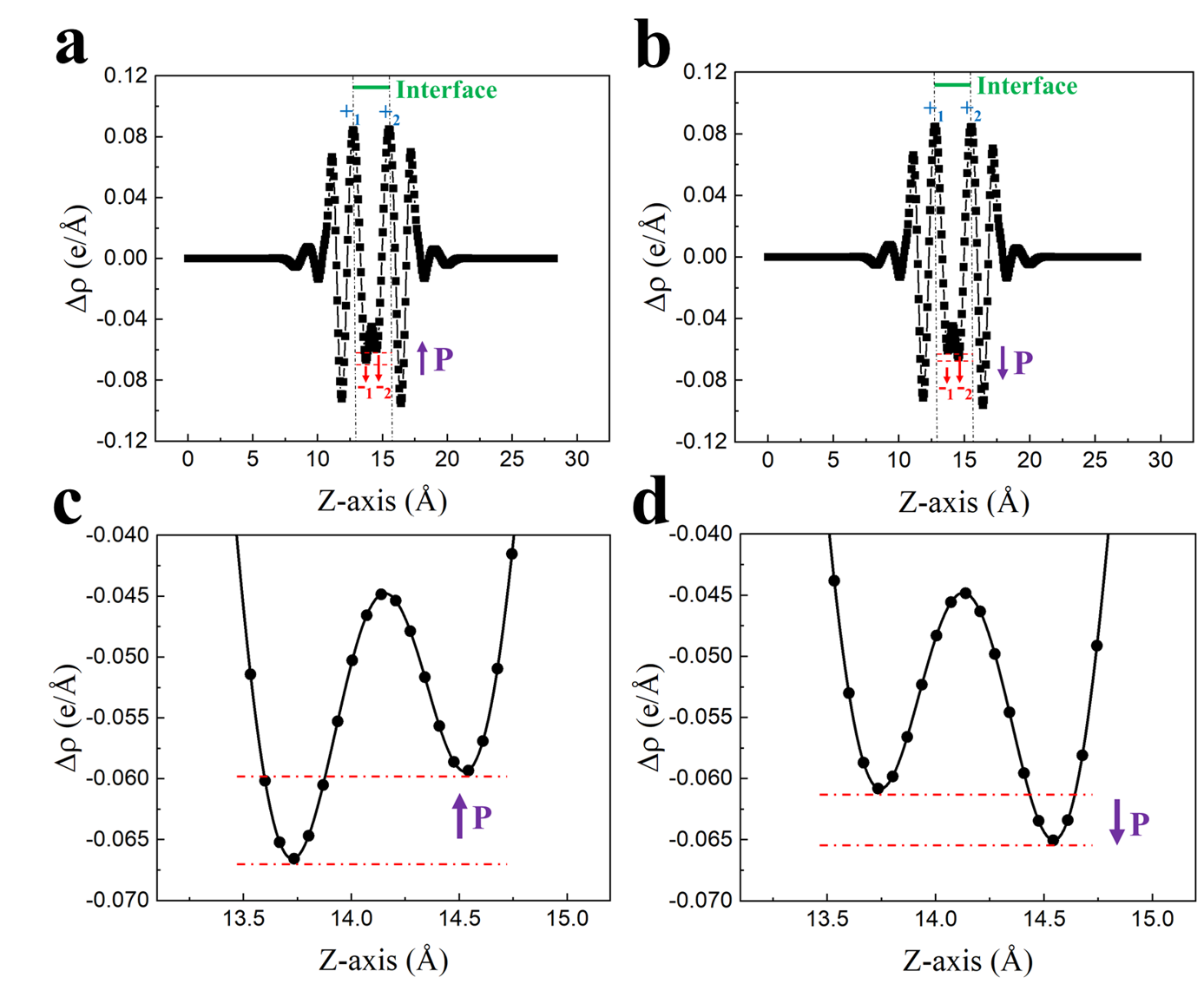
**

**Figure S8.** (a, b) One-dimensional layer-averaged differential charge density (Δρ) along the out-of-plane (z) axis for bilayer 1T′-WTe₂ with interlayer sliding of +2.8% and −2.8%, respectively. Charge accumulation and depletion near the interface (green) indicate interlayer charge transfer and net dipole formation. (c, d) Zoom-in views of the interfacial region corresponding to panels (a) and (b), respectively. The shift in the charge density minima between (c) and (d) confirms the reversal of interfacial polarization direction

**S9 DFT calculations for layer-number-dependent polarization**

To clarify the layer-number dependence of interfacial polarization, we emphasize that the emergence of net out-of-plane electric polarization in 1T'-WTe₂ multilayers is governed by the parity of the layer number. While our DFT analysis explicitly treats bilayer and trilayer configurations, the physical mechanism extends to thicker even-layered systems. Specifically, in even-layer 1T'-WTe₂ (e.g., 2L, 4L, 6L), inversion symmetry is broken due to the Pm space group, and interfacial dipoles do not fully cancel, resulting in a finite net polarization. In contrast, odd-layer structures (e.g., 3L, 5L) possess global inversion symmetry (P2₁/m) such that the interfacial dipoles are arranged in an antiparallel fashion and cancel each other. This symmetry-dependent dipole cancellation is validated by the experimentally observed SHG contrast, which appears exclusively in even-layer regions, and further supported by PFM imaging. Therefore, although the DFT calculations focus on lower-order structures, the trend of parity-driven polarization is intrinsic to the stacking symmetry and remains valid for multilayer systems, providing a layer-number-dependent but symmetry-mediated framework for tunable interfacial ferroelectricity.

**S10 Methods**

**CVD reactions of the 1T’-WTe_2_**

A home-built CVD system was used to synthesize few-layer 1T’-WTe_2_ films. A SiO_2_/Si substrate was placed facing down on the ceramic crucible that is filled with 40 mg of WO_3_ powders (Alfa Aesar, 99.8%), 10 mg of KCl powders (Duksan Pure Chemicals, >99.0%), and 0-10 mg Te powders (Acros Organics, 99.8%). Another ceramic crucible holding the Te powders (100 mg) was located upstream at a distance of 9 cm away from the crucible for the mixtures of precursors and promoters. The furnace was heated to 700 °C for 10 min and maintained for 3 min for growth reactions. The gaseous flows of argon and hydrogen were 40 and 15 sccm, respectively. Crystal growth of the 1T’-WTe_2_ is mainly along [100] direction with a ribbon-like shape because of anisotropic surface energy^2^. To increase the domain size, we applied additional Te powder into the central crucible, which contained the mixed powder of WO_3_ and KCl. A mixture of alkali metal chlorides (e.g., NaCl and KCl) and WO_3_ can form metal oxychlorides, further reducing the metal precursors' melting points in a hydrogen-involved atmosphere. Essential Te concentration is required in the initial growth stage due to the relatively low vapor pressure and low chemical reaction activity of Te with W.^3^

**Manipulation of synthetic materials**

A water-assisted transfer process was adopted to manipulate the synthetic 1T’-WTe_2_ films for further measurements, including TEM and scanning probe microscopy (SPM). After the synthetic samples were gently immersed in de-ionized water, a continuous layer of the WTe_2_ was peeled off from the substrate due to the surface tension of the water.

**TEM measurements**

Atomic structures were observed using spherical-aberration corrected field-emission TEM (JEM-ARM200FTH, JEOL) operating at an accelerating voltage of 80 kV.

**SPM characterization**

SPM measurements were performed using Dimension ICON (Bruker Ltd.) at room temperature (RT). We used Tap300Al-G with a force constant of ~40 N/m for the AFM measurements and PPP-EFM (silicon probe with PtIr_5_ coating) with a force constant of ~2.8 N/m and the applied alternating current (AC) voltage of 2 V in **Figure 2b** for the PFM measurements.

**Raman spectroscopy measurements**

Polarization-resolved Raman spectroscopy measurements were carried out with a confocal Raman microscope (ProTrustech Co., Ltd.) using a 532 nm excitation laser wavelength. The laser power was kept as low as 0.5 mW to avoid possible damage. The scattered Raman signal was collected by a 50x microscope objective and dispersed with a blazed grating of 1800 gr/mm. A polarizer sets the polarization state of incoming light while the scattered light passes through the same objective, and the analyzer aligns parallel to the polarization of the incident laser. Samples were mounted on a rotator for variable angle Raman measurements. The Si peak was set at 520 cm^-1^ and used for calibration in the analyses. All Raman spectra were acquired at ambient conditions.

**SHG measurements**

SHG measurements were carried out with a Leica SP5 MP confocal microscope working at nondescan mode. A mode-lock Ti-Sapphire laser with tunable broadband wavelength (700 to 1000 nm) was sent through a linear polarizer to increase the extinction ratio and directed to a microscope with a 20x objective. Then, we collected the reflected signal with a linear polarized analyzer set in the direction of the incident laser. A galvo-mirror-based scanning system (excitation at 950 nm, SHG at 475 nm) with a 20× objective lens (NA ≈ 0.4), resulting in an estimated spatial resolution of sub-1 µm. Samples were mounted on a rotator for variable angle SHG measurements. The SHG signal was extracted from the fundamental beam by a 680 nm short pass filter (Semrock) and a 495 nm beam splitter and detected with a photomultiplier detector. With the pulse repetition rate of 76 MHz and the highly sensitive photomultiplier detector, we can quickly obtain data from the average pixel intensity of SHG images. The SHG intensity difference between even- and odd-layer regions is significantly higher than the background level, ensuring a high signal-to-noise ratio and reliable identification of ferroelectricity in the synthetic WTe_2_.

**Simulated diffraction patterns of 1T’-WTe_2_**

CrystalMaker and SingleCrystal (CrystalMaker Software Ltd.) were used to simulate the crystal structure and diffraction patterns (**Figure 1c,d**).

**First-principles DFT calculations**

The band structures of bilayer 1T’-WTe_2_ were computed using the projector augmented wave method as implemented in the VASP package^4-6^ within the GGA scheme.^7^ The lattice structure was optimized using a 21 × 11 × 1 Monkhorst-Pack k-point mesh. The optimized atomic forces are less than 1 meV/$Å$. Free-standing slabs simulated the WTe_2_ films with a vacuum thickness larger than 20 $Å$ separating the slabs. The spin-orbit coupling (SOC) was included self-consistently in all the calculations. Regarding the calculation methods for dipole moment and polarization, according to reference^18^ by utilizing charge density, the polarization with dipole correction was calculated by the following formula: $P=\frac{e}{A}\int\left( r-R_{ref} \right)\rho(r)d^{3}r$ , where $A$ is the area of the slab model, $\rho$ is the charge density, and $R_{ref}$ is the reference point at the center of the slab system. The Van-der Waals dispersion correction (IVDW = 12) was considered in all calculations.

| Layer Number | Inversion Symmetry | Net Polarization | Polarization Behavior |
| --- | --- | --- | --- |
| 2L | Broken | ±0.12 pC/m | Nonzero; switchable via interlayer sliding |
| 3L | Preserved | 0 | Dipoles cancel; no net polarization |
| 4L | Broken | ±0.12 pC/m | One uncompensated dipole remains |
| 5L | Preserved | 0 | Antiparallel dipoles cancel |
| 6L | Broken | ±0.12 pC/m | Same behavior as 4L; net interfacial polarization |

**Table S3.** DFT calculations for layer-number-dependent polarization *of* the 1T’-WTe_2_

|  | **Even** layers (2n, n󠄀∈N) | **Odd** layers (2n+1, n∈N) |
| --- | --- | --- |
| **T_d_** phase | 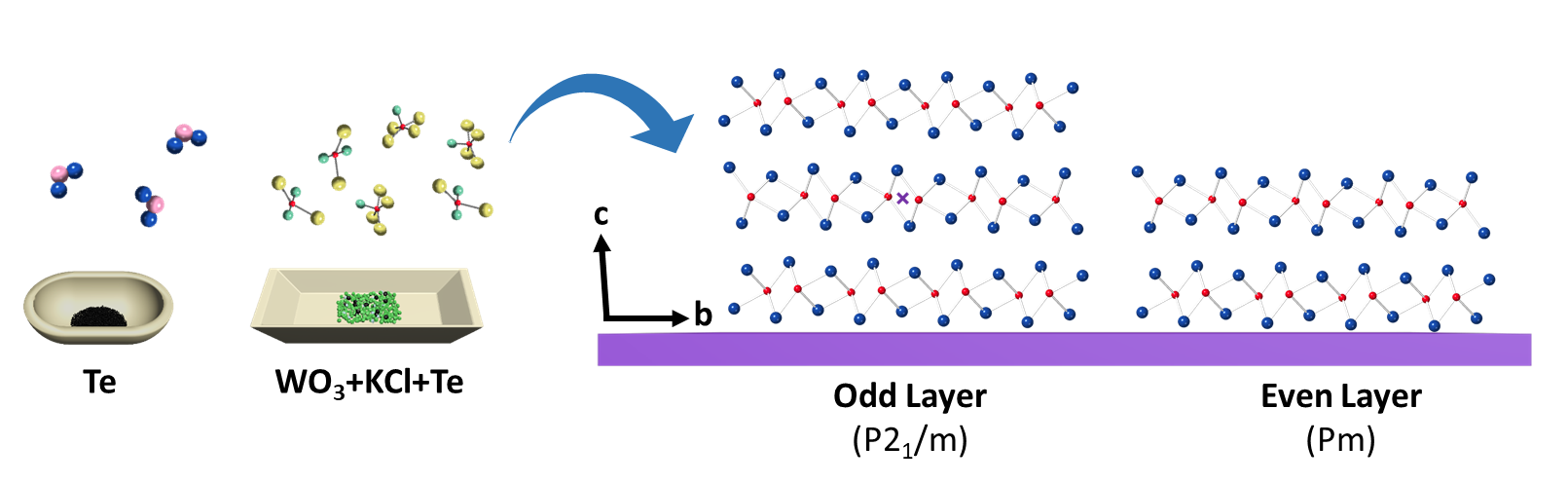Pm #6 | 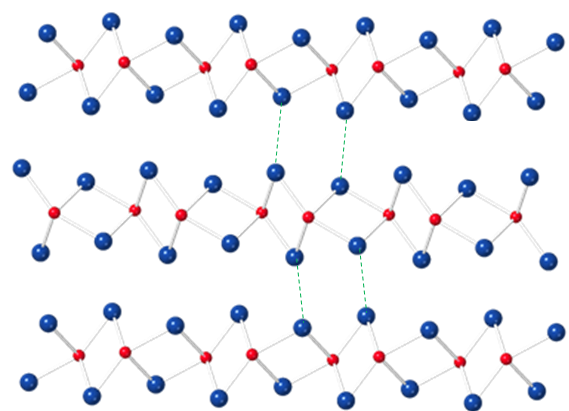 Pm #6 |
| **1T’** phase |  | 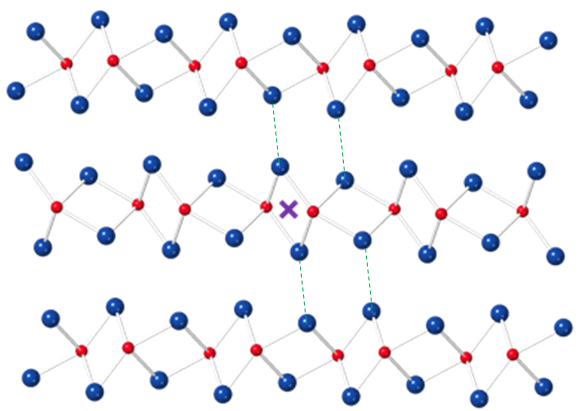 P2_1_/m #11 |

**Table S1.** Schematic comparison of difference between T_d_- and 1T’-WTe_2_. Note that the violet cross mark indicates an inversion symmetry center.

| Atom | Site | X | y | z |
| --- | --- | --- | --- | --- |
| W | 2(e) | 0.183 | 0.250 | 0.007 |
| W | 2(e) | 0.318 | 0.750 | 0.507 |
| Te | 2(e) | 0.589 | 0.250 | 0.104 |
| Te | 2(e) | 0.097 | 0.750 | 0.149 |
| Te | 2(e) | 0.557 | 0.750 | 0.352 |
| Te | 2(e) | 0.056 | 0.250 | 0.396 |

**Table S2.** Crystallographic data for the 1T’-WTe_2_. Space group P2_1_/m, monoclinic. Lattice parameters a = 6.330, b = 3.469, c = 14.070 Å, β = 93˚55’.^1^

**Reference**

1. W. G. Dawson, D. W. Bullett. Electronic structure and crystallography of MoTe_2_ and WTe_2_. *Journal of Physics C: Solid State Physics* **1987**, 20, 6159.
2. Xu, M.; Tang, B.; Lu, Y.; Zhu, C.; Lu, Q.; Zhu, C.; Zheng, L.; Zhang, J.; Han, N.; Fang, W.; Guo, Y.; Di, J.; Song, P.; He, Y.; Kang, L.; Zhang, Z.; Zhao, W.; Guan, C.; Wang, X.; Liu, Z. Machine Learning Driven Synthesis of Few-Layered WTe2 with Geometrical Control. Journal of the American Chemical Society 2021, 143 (43), 18103-18113.
3. Zhou, J.; Liu, F.; Lin, J.; Huang, X.; Xia, J.; Zhang, B.; Zeng, Q.; Wang, H.; Zhu, C.; Niu, L.; Wang, X.; Fu, W.; Yu, P.; Chang, T.-R.; Hsu, C.-H.; Wu, D.; Jeng, H.-T.; Huang, Y.; Lin, H.; Shen, Z.; Yang, C.; Lu, L.; Suenaga, K.; Zhou, W.; Pantelides, S. T.; Liu, G.; Liu, Z. Large-Area and High-Quality 2D Transition Metal Telluride. Advanced Materials 2017, 29 (3), 1603471.
4. Blöchl, P. E. Projector augmented-wave method. Physical Review B 1994, 50 (24), 17953-17979.
5. Kresse, G.; Joubert, D. From ultrasoft pseudopotentials to the projector augmented-wave method. Physical Review B 1999, 59 (3), 1758-1775.
6. Kresse, G.; Furthmüller, J. Efficiency of ab-initio total energy calculations for metals and semiconductors using a plane-wave basis set. Computational Materials Science 1996, 6 (1), 15-50.
7. Perdew, J. P.; Burke, K.; Ernzerhof, M. Generalized Gradient Approximation Made Simple. Physical Review Letters 1996, 77 (18), 3865-3868.
